# Pharmacologic targeting of β-catenin improves fracture healing in old mice

**DOI:** 10.1038/s41598-019-45339-0

**Published:** 2019-06-21

**Authors:** Yoon Hae Kwak, Tomasa Barrientos, Bridgette Furman, Hazel Zhang, Vijitha Puviindran, Hattie Cutcliffe, Jonas Herfarth, Eugene Nwankwo, Benjamin A. Alman

**Affiliations:** 1 Department of Orthopaedic Surgery, Duke University, Durham, NC USA; 2 Present Address: Division of Orthopaedic Surgery, Severance Children’s Hospital, Yonsei University College of Medicine, Seoul, South Korea; 3 Regeneration Next, Duke University, Durham, NC USA

**Keywords:** Molecular medicine, Target identification

## Abstract

β-catenin protein needs to be precisely regulated for effective fracture repair. The pace of fracture healing slows with age, associated with a transient increase in β-catenin during the initial phase of the repair process. Here we examined the ability of pharmacologic agents that target β-catenin to improve the quality of fracture repair in old mice. 20 month old mice were treated with Nefopam or the tankyrase inhibitor XAV939 after a tibia fracture. Fractures were examined 21 days later by micro-CT and histology, and 28 days later using mechanical testing. Daily treatment with Nefopam for three or seven days but not ten days improved the amount of bone present at the fracture site, inhibited β-catenin protein level, and increased colony forming units osteoblastic from bone marrow cells. At 28 days, treatment increased the work to fracture of the injured tibia. XAV939 had a more modest effect on β-catenin protein, colony forming units osteoblastic, and the amount of bone at the fracture site. This data supports the notion that high levels of β-catenin in the early phase of fracture healing in old animals slows osteogenesis, and suggests a pharmacologic approach that targets β-catenin to improve fracture repair in the elderly.

## Introduction

Almost a third of humans will fracture a bone, and most often these injuries go on to successfully heal. However, a lack of healing (fracture nonunion) is estimated to occur in over 5% of injuries, depending on the anatomic location^1^. Effective bone regeneration relies on the inflammatory response to tissue injury and the physiology of bone development. Various environmental and biological factors can hinder this regenerative process^2^. With age, the pace of fracture repair slows, and the risk of non-union increases^3,4^. This slower pace of repair in older individuals is responsible for increased morbidity and even mortality^5^.

While there are many factors that could impair fracture healing with aging, the pace of fracture repair can be rejuvenated by circulating factors present in young animals^6^. Recent data suggests that factors produced by young macrophage cells pay a critical role in the rejuvenation process^7^. While these factors regulate the pace of repair, their effects on mesenchymal cells differentiating to osteoblasts are mediated by signaling pathways such as β-catenin. β-catenin signaling plays different roles in mesenchymal differentiation at different repair stages. In the initial phase of repair, the level of β-catenin needs to be precisely regulated as levels that are too high or too low will prevent undifferentiated mesenchymal cells from becoming osteochondral progenitors and will inhibit fracture healing. Once the cells differentiate to osteochondral progenitors, higher levels of β-catenin stimulate osteogenesis and enhance fracture repair^8,9^.

Data from studies in mice shows that older animals have a higher level of β-catenin protein during the early phase of fracture repair. Genetic inhibition of β-catenin activation rejuvenates the fracture phenotype in older mice^6^. This is in contrast to data from young mice, in which inhibition of β-catenin impairs fracture healing^8^. Since β-catenin protein level regulates the capacity of undifferentiated mesenchymal cells to become osteochondral progenitors, an elevated protein level in older animals will prevent this differentiation and instead cells will maintain a fibroblastic like phenotype. Such a cell type will not contribute to normal fracture repair. Thus, in older animals but not younger animals lowering β-catenin protein level at the initial phases of repair will increase the proportion of undifferentiated mesenchymal cells at the fracture that can go on to become osteoblasts and heal the injury. In the later phases of the repair process, β-catenin is required for osteoblastic differentiation. Targeting β-catenin to effectively improve healing in old animals would need to focus on the earlier phases of the repair process.

The ability of pharmacologic agents to improve fracture repair by targeting β-catenin in is not known. Nefopam is one agent that is capable of targeting β-catenin in mesenchymal cells^10^. It is a non-opioid analgesic of the benzoxazolcine family of compounds. This agent has minimal side effects and is used in clinical settings across Europe and Asia^11^. Although the mechanisms of action of Nefopam is not fully understood, its use decreased β-catenin levels in hypertrophic scar and desmoid tumors^10^. Another class of agents that can inhibit β-catenin are tankyrase inhibitors. XAV939 binds the tankyrase catalytic poly-ADP-ribose polymerase (PARP) domain, and subsequently increases β-catenin destruction through stabilization of Axin. It has been utilized as an inhibitor of β-catenin *in-vivo*^12–15^. Here we examined the ability of Nefopam and XAV939 in the early phases following a fracture to improve fracture repair in elderly mice.

## Results

### Nefopam treatment stimulates ossification during fracture repair in 20 month old mice

**Figure 1:**
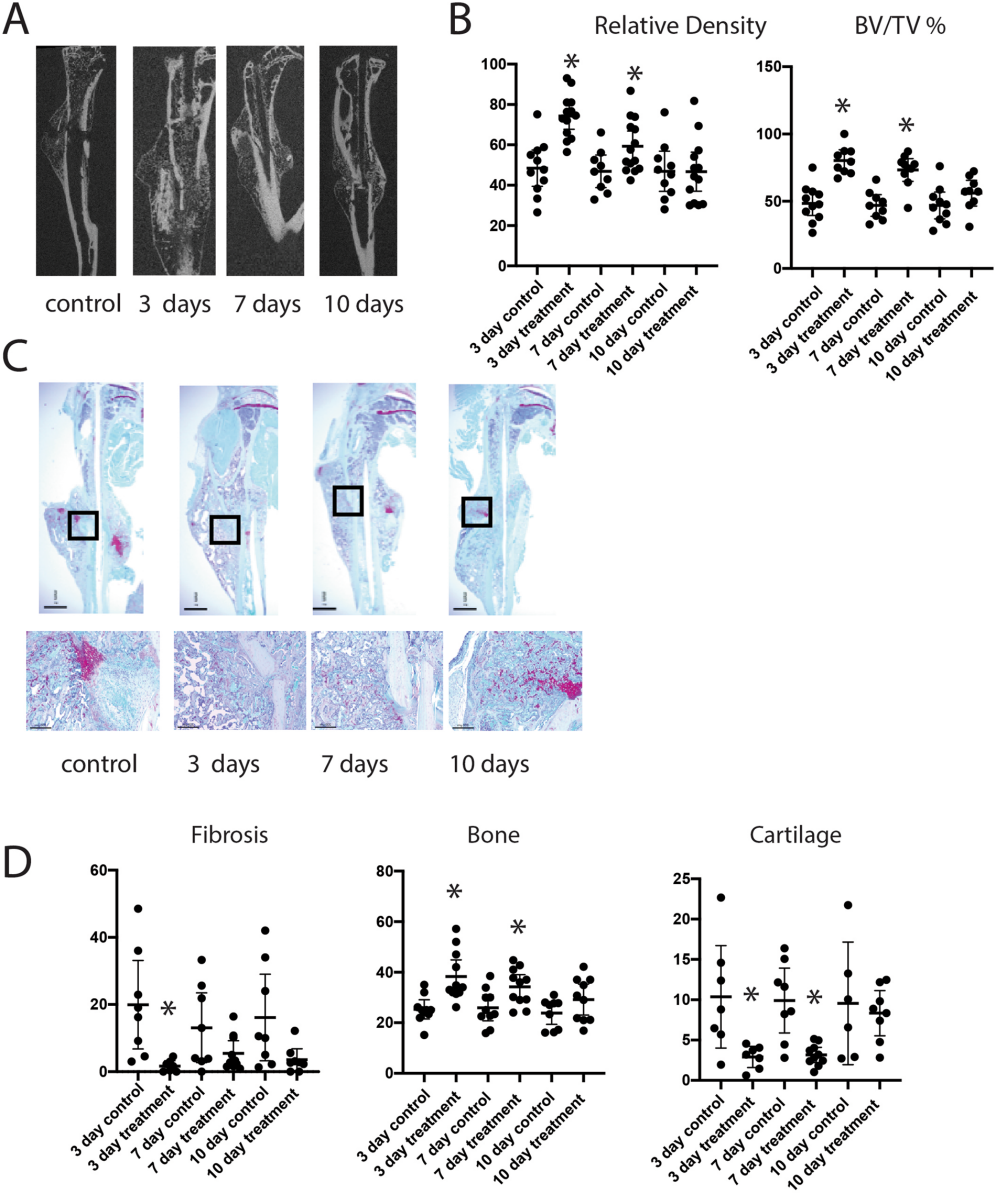
Nefopam treatment increases osteogenesis during fracture healing in old mice. (**A**) Representative micro-CT images of healing tibia fractures 21 days after fracture was generated. (**B**) Graphical representation of relative density or bone volume/total volume (BV/TV) in %. Each data point is shown as well as means and 95% confidence intervals. An asterisk over a data point indicates a significance *p* <  0.05 compared to control, comparison using ANOVA. (**C**) Representative histologic images from the tibia of an old mouse 21 days after fracture. (**D**) Graphical representation of percent fibrosis, cartilage, or bone. Each data point is shown as well as means and 95% confidence intervals. An asterisk over a data point indicates a significance *p* <  0.05 compared to controls, comparison using Kruskal-Wallis.

To determine if pharmacologic modulation of β-catenin might improve fracture repair in older animals, twenty-month-old male mice were treated with nefopam after generating a tibia fracture. The inflammatory phase of fracture repair in mice occurs during the first three days, and the initially proliferative phase when osteochondral progenitors are present occurs at 7 days. Previous studies in mice found that β-catenin protein levels were increased at 3 and 7 days following a fracture, and returned towards baseline levels by 10 days^9^, The 3 and 7 day time frame is also the period in which β-catenin protein levels are even higher in older animals^6^. Thus, we selected time frames to test the drug when we knew β-catenin protein levels were elevated, as well as one longer duration time point (treatment with nefopam from 3 to 10 days). Treatment was carried out using a dose known to inhibit β-catenin in mesenchymal cells^16^. Control mice were treated with carrier alone for the same time interval. At 21 days after fracture, the mice were sacrificed and bones analyzed histologically and by μCT. There were no significant differences in μCT parameters, histomorphometric parameters, or mechanical properties for the carrier controls treated for 3, 7, or 10 days. Treatment with Nefopam for 3 or 7 days improved the bone volume/total volume and the relative density at the fracture site, while mice treated with Nefopam for 10 days displayed little difference compared to controls (Fig. 1A,B). Histomorphometry was undertaken from sections from the fracture calluses stained with Safranin O/Fast Green. Three days of Nefopam treatment resulted in an increase in bone at the fracture site and a decrease in fibrotic tissue. With seven days of treatment there was an increase in bone as well, but not a significant change in fibrous tissue proportion compred to controls. There was not a significnat difference between ten day treated mice and controls (Fig. 1C,D). Thus, short term but not longer term treatment increased ossification at the fracture site. We did not observe any change in μCT parameters in the unfractured bone (supplementary data). This is not unanticipated, given the short duration of drug therapy. There were no side effects observed in these mice as well, and there was no difference in the weight of the mice between treated and control animals.

### Nefopam decreases β-catenin protein level and increases osteoblastic differentiation in old mice

**Figure 2:**
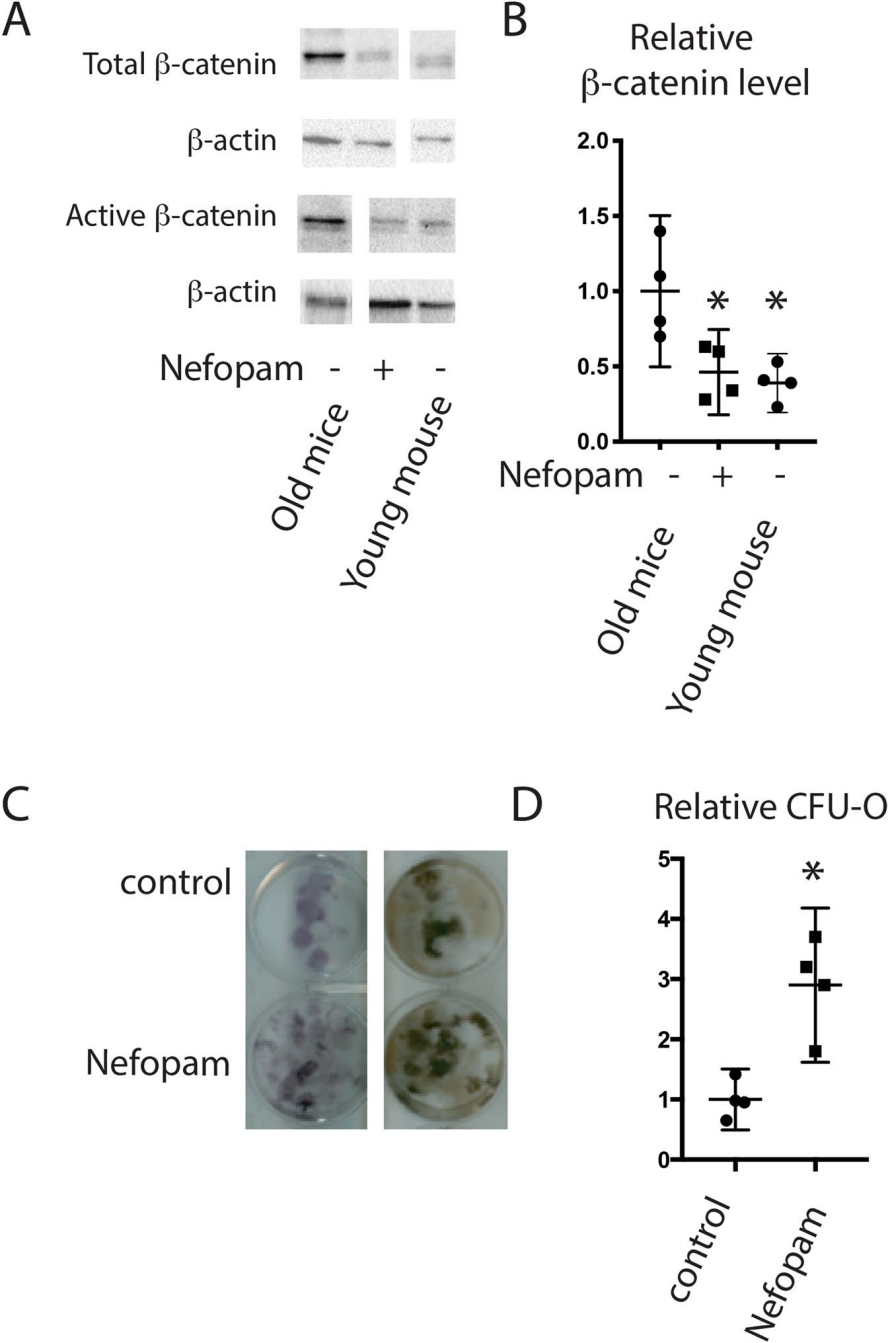
Nefopam modulates β-catenin during fracture repair and increases colony forming units osteoblastic in old mice. (**A**) Representative Western blot for β-catenin, active β-catenin, and β-actin as a loading control from protein extracts from healing tibia fractures in 20 month old mice treated with nefopam or control, or young mice as a reference. Each set of bands for each protein is from a single blot, with the full blot shown in supplementary data. The loading control is from a stripped and probed membrane. (**B**) Graphical representation of relative β-catenin level, with controls arbitrarily averages to “1.” Each data point is shown as well as means and 95% confidence intervals. An asterisk over a data point indicates a significance *p* <  0.05 compared to controls, comparison using. (**C**) Representative Colony forming units osteoblastic (CFU-O) from bone marrow from mice treated with nefopam or controls. (**D**) Graphical representation of relative CFU-O, with controls arbitrarily averages to ‘1.” Each data point is shown as well as means and 95% confidence intervals. An asterisk over a data point indicates a significance *p* <  0.05 compared to controls, comparison using t-test.

Nefopam might alter fracture repair without regulating β-catenin. We thus examined protein expression *in-vivo* and osteoblast differentiation *in-vitro*. Western blot analysis was employed to quantify levels of β-catenin protein and activated β-catenin protein. Data was compared with fracture callous in young, 4 month old, mice. There was an increase in total and active β-catenin protein in callouses from old mice, and treatment with nefopam for three days substantially reduced the total and active β-catenin protein level in calluses from old mice (Fig. 2A,B). Colony forming units osteoblastic (CFU-O) derived from 20 month old mice were evaluated to investigate osteoblast differentiation after Nefopam treatment. Bone marrow from tibiae were flushed and plated for culture following 3 days of Nefopam treatment. There was a significantly greater number of CFU-O in cultures from mice treated with Nefopam compared to controls (Fig. 2C,D). There was also a higher level of expression of genes important in osteogenesis (supplementary data). Taken together, this supports the notion that Nefopam exerts its effects by modulating β-catenin to the optimal level to stimulate mesenchymal cells differentiating to osteoblasts.

### Tankyrase inhibition increases ossification after fracture in 20 month old mice

**Figure 3:**
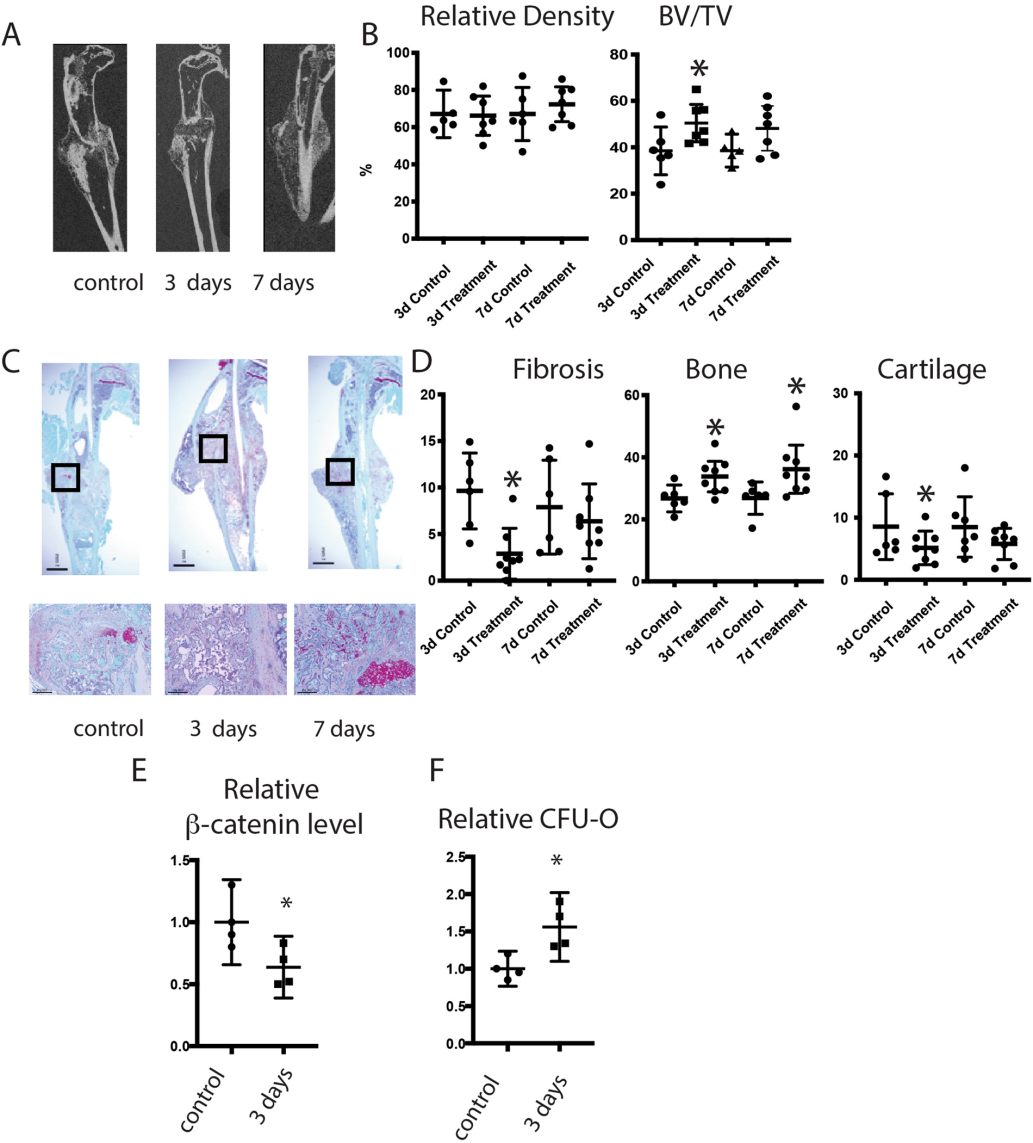
Tankyrase inhibition increases osteogenesis during fracture healing in old mice. (**A**) Representative micro-CT images of headlining tibia fractures 21 days after fracture. (**B**) Graphical representation of relative density or bone volume/total volume (BV/TV) in %. Each data point is shown as well as means and 95% confidence intervals. (**C**) Representative histologic images from the tibia of an old mouse 21 days after fracture. (**D**) Graphical representation of percent fibrosis, cartilage, or bone. Each data point is shown as well as means and 95% confidence intervals. An asterisk over a data point indicates a significance *p* <  0.05 compared to controls.

To determine if other agents that inhibit β-catenin might regulate fracture healing in old mice, we investigated the tankyrase inhibitor XAV939. Mice were treated for 3 or 7 days after fracture with a previously established effective dose^13,15^, and compared to carrier controls 21 days after fracture. μCT analysis showed that XAV939 treatment did not significantly increase ossification (Fig. 3A,B). Histomorphometry, however, showed a significant increase in bone and a decrease in fibrous tissue at the fracture sites (Fig. 3C,D). We then analyzed β-catenin protein levels and the ability of mice to form CFU-O. XAV939 had a more modest effect on β-catenin and CFU-O compared to nefopam treatment (Fig. 3F,F). This data is consistent with the notion that the less effective ability of XAV939 to lower β-catenin is the reason that the effect on fracture repair was relatively modest. Because drugs might have deleterious systemic effects on which might alter fracture repair, we examined the weight of the mice and observed them for signs of distress. There was no evidence of distress or side effects in the mice, and no difference in weight between the treated mice and controls. Tankyrase inhibitors might inhibit osteoclasts^17^, and we analyzed TRAP stained cells in the various mice. There was no difference in osteoclast numbers in treated mice compared to controls (supplementary data).

### Nefopam treatment improves the work to failure in healing tibia fractures in old mice

**Figure 4:**
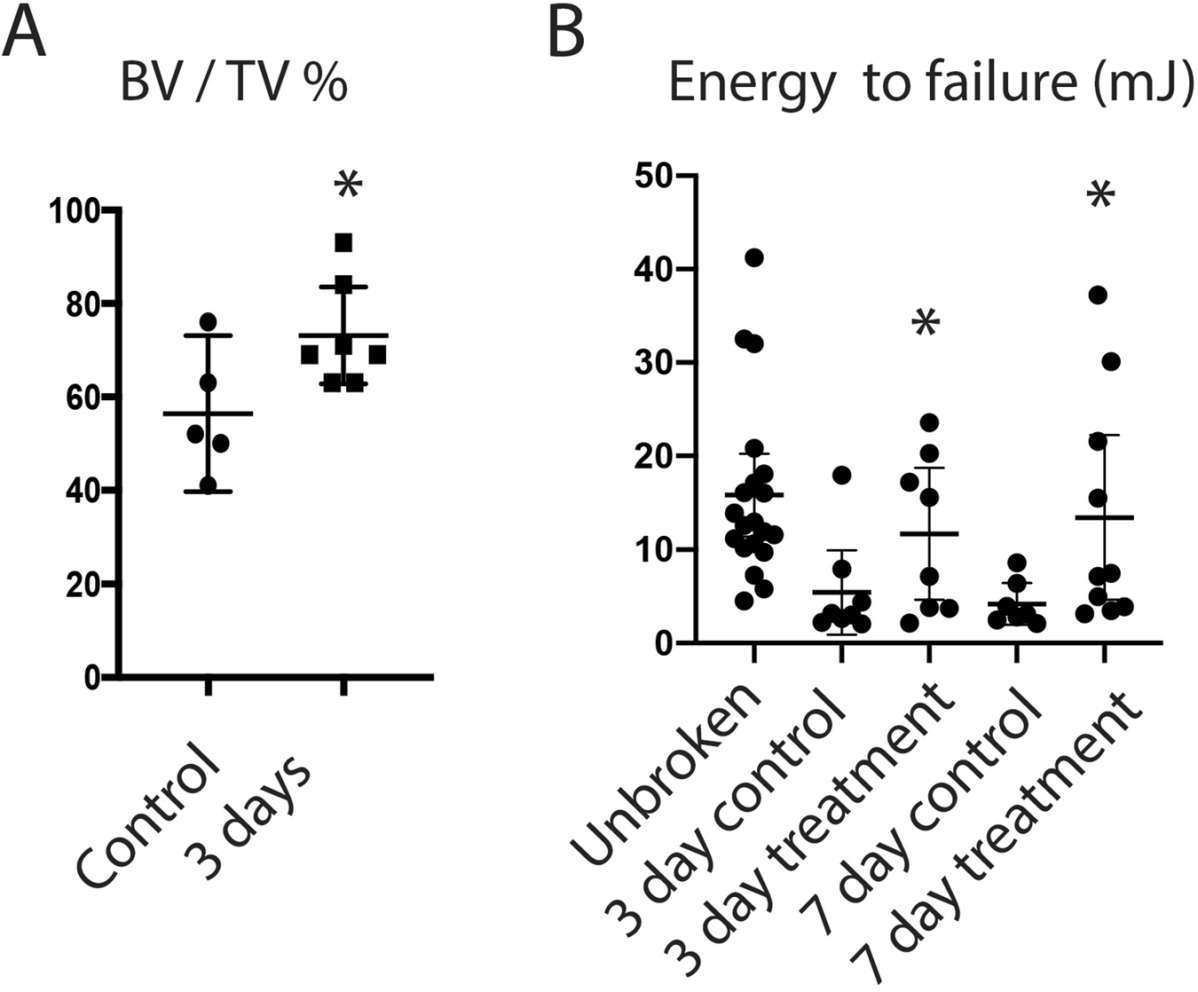
Nefopam treatment improves mechanical properties of healing fractures in 20 month old mice. (**A**) Graphical representation of bone volume/total volume (BV/TV) in % for mice 28 days after a fracture. Each data point is shown as well as means and 95% confidence intervals. An asterisk over a data point indicates a significance *p* <  0.05 compared to control, comparison using t-test. (**D**) Graphical representation of work to fracture of two different durations of treatment. Each data point is shown as well as means and 95% confidence intervals. An asterisk over a data point indicates a significance *p* <  0.05 compared to controls, comparison using Kruskal-Wallis.

The ultimate goal of fracture repair is to reconstitute the mechanical properties of the injured bone. To determine if Nefopam treatment would improve the mechanical properties of the fractured bone, we measured the work to failure 4 weeks after injury and compared this to unfractured bone. There was a significant decline in work to failure in the healing control bone compared to uninjured bone. However, there was an increase in the work to failure in healing fractures in bone from Nefopam treated mice (Fig. 4). The work to failure in the healing bone from Nefopam treated mice approached that of the uninjured tibia. The observed increased bone density in healing fractures in Nefopam treated mice results in a significant increase in bone strength as well.

## Discussion

Prior work suggests that high levels of β-catenin in older animals impairs osteoblastic differentiation^6^. Our data supports this notion but also shows that short-term, but not longer term, β-catenin inhibition improved the quality of bone repair in older mice. Nefopam is an agent used in patients with an established safety record^10,11^. Here we identified a potential repurposed use for a drug in bone repair in situations in which β-catenin protein levels are elevated, and show that agents that target β-catenin may serve as a novel pharmacologic approach to improve healing in the elderly.

XAV939 also will target β-catenin. XAV939 treatment did not have a strong an effect on either β-catenin or on osteogenesis during fracture repair as Nefopam. This supports the notion that targeting β-catenin is the mechanism of action for improving fracture repair. Inactivation of tankyrase also targets osteoclasts and can produce low bone density. XAV939 did not have an effect on osteoclast numbers, and likely the duration of use was too short to result in a phenotypic change. While toxicity is a concern when inhibiting β-catenin signaling pathway^18,19^, both nefopam and XAV939 are reported to have mild side effects, At the doses we utilized for the short duration, side effects such as weight loss and diarrhea were not observed in the animals.

Similar to the situation in aging, mice lacking *Nf1* also exhibit high levels of β-catenin in the early phases of healing fractures^20^. Short term nefopam treatment in mice lacking *Nf1* improves ossification at the fracture site^16^. In these studies, nefopam was also used for short periods of time in young control mice, and these mice did not show any difference in the fracture repair phenotype^16^. Our finding in aging, extends the role of nefopam as a modulator of osteoblastic differentiation and bone fracture repair in instances in which β-catenin protein is elevated. Aging and neurofibromatosis are two such instances.

One potential limitation of this study is that we examined only male mice. However, previous work found no differences in fracture repair parameters between male and female 20 month old C57BL6 mice^6,7^. We are undertaking a variety of studies examining repair in older animals in our research group, including parabiosis work. Because female mice are preferable for parabiosis work, as they adjust to the conditions better than male mice^6^, we preferentially used the female mice for that work. In order to minimize the number of animals bred for this research, and because there are no reported differences in repair parameters between male and female mice, we used male mice from our colony for this work.

The data in this study suggest a possible pharmacologic approach to the rejuvenation of fracture healing in old mice via modulation of β-catenin. Short term inhibition during the initial phases of fracture healing, a time when undifferentiated mesenchymal cells can differentiate to osteochondral progenitors, is most effective. Such a non-invasive, short term pharmacologic approach with low side effects has obvious advantages over more invasive approaches to stimulate fracture healing. While we used injection of the drugs in this study to better control dosing, nefopam and tankyrase inhibitors are available as oral agents, and could be administered via an oral route in a clinical trial.

## Materials and Methods

### Mice and treatments

Animal protocols were approved by the Institutional Animal Care and Use Committee (IACUC) at Duke University. Experiments were performed in compliance with NIH guidelines on the use and care of laboratory and experimental animals. C57BL6 mice were provided from Charles River Laboratories (Wilmington, MA, USA) and housed under a 12-hour light, 12-hour dark cycle. Food and water were provided *ad libitum*. Male mice were used for the study based on our previous data showing that old male and female C57BL6 mice have identical fracture repair characteristics^6,7^. Nefopam hydrochloride was purchased from MedChemExpress (Monmouth Junction, NJ, USA) and dissolved in normal saline as carrier control, then delivered by intraperitoneal injection (i.p.) (30 mg/kg). XAV939 was purchased from Selleck (Houston, TX, USA) and dissolved in 4% dimethyl sulfoxide (DMSO). Corn oil was used for vehicle or carrier control. XAV939 was subsequently delivered by i.p. injection (20 mg/kg). Mice were randomly selected to either receive treatment or carrier. Treatment was started from the day of surgery and progressed at a duration of 3, 7, or 10 days. Doses were selected from prior studies. Animals were observed for any deleterious symptoms and their weight was recorded before, during and after treatment.

### Fracture generation

A tibia fracture with intramedullary stabilization was used to study bone repair^8^. Briefly after anesthesia was induced, the surgical area was shaved and disinfected. A 3-mm longitudinal incision was made in the patella tendon and 0.5mm hole was drilled by a 25G syringe needle above the tibial tuberosity. Intramedullary fixation was achieved using a 0.7 mm insect pin then the mid-tibial diaphysis was transected with small surgical scissors. Osteotomies were made parallel to the tibial plateau. The incision was then closed using sutures. Analgesia was provided to all mice. As in our prior work^6–8,16,20^. Slow release buprenorphine hydrochloride analgesia (0.03 mg/kg). was provided as an analgesic during the generation of a tibial injury and the initial painful period after the fracture was generated until animals returned to their usual activity level.

### Radiograph and μCT analyses

The fractured tibia, as well as, and the intact, contralateral tibiae were studied after mice were sacrificed at postoperative 21 days. Tibiae were then fixed in 10% formalin for 48 h at room temperature (25 °C) and then transferred to 70% ethanol. Implants were removed from the tibiae prior to scanning. The quality of fracture repair was assessed using the Faxitron MX20 X-ray system (Faxitron, Tucson, AZ, USA). Quantitative three-dimensional evaluation of the fracture callus and healing was undertaken by μCT using a Viva CT 80 (Scanco, Brüttisellen, Switzerland) at 55 kVp and 145 μA with a resolution of 15.6 μm voxel size. A hydroxyapatite calibration phantom was used to scale values of linear attenuation for the calcified tissues to bone density values (mg/cm^3^). Morphometric parameters were quantified^21^ at the site of fracture which included 1mm proximal and distal from the fracture location. The original grayscale image datasets of the tibia region of interest were imported into the manufacturer software and the cortical and callus regions were separated using an automated contouring method. Calcified tissues were segmented from soft tissues using a global thresholding procedure with a threshold of 480 mg HA/cm^3^, which represents 45% of the attenuation of mature cortical bone (*19, 20*). Callus maturity was measured using differential thresholding in which immature bone was defined by a threshold of 169 to 400 mg HA/cm^3^. As an additional analysis, the unfractured tibia was also examined using μCT. Parameters such as Bone volume/total volume in the tibia were analyzed using ASBMR guidelines^22^.

### Histology and histomorphometry

After the μCT imaging process, tibiae were decalcified in 14% EDTA (pH 7.4) for 7 days at room temperature. Decalcified samples were dehydrated in a graded ethanol series, embedded into paraffin. Sections 5 µm thick were prepared and stained using Safranin-O (to stain proteoglycans/cartilage red) and counterstained with Fast Green (to stain bone green/blue). For evaluation of osteoclastogenesis, the formation of tartrate-resistant acid phosphatase (TRAP)-positive multinucleated cells was visualized by TRAP staining (Sigma-Aldrich). The sections were examined and photographed using a Leica digital imaging system (Leica, Wetzlar, Germany). Histomorphometry measurements were quantified using ImageJ software (National Institutes of Health, USA).

### Protein and RNA analysis

Bone fragments were collected from the tibia from mice treated with carrier, Nefopam, or XAV939, harvested on the day 3 of treatment. After dissection, the harvested tissues were immediately frozen in liquid nitrogen, pulverized then homogenized in radioimmunoprecipitation assay (RIPA) buffer (Thermo Fisher Scientific). Total protein was quantified with Bradford reagent using a bovine serum albumin (BSA) standard curve as a reference. SDS-polyacrylamide gels at 8% concentration were loaded with 25 µg of protein lysate. Anti-β-catenin antibody (AB19022) was purchased from Millipore, an antibody that recognizes activated (unphosphorylated at Ser-37 and Thr-41) β-catenin (clone 8E7) was also obtained from Millipore and an anti-β-actin (13E5) antibody was purchased from Cell Signaling and was probed as a loading control. Indicated antibodies were detected using horseradish peroxidase-conjugated secondary antibodies and ECL chemiluminescence (Bio-Rad). Protein expression was analyzed by measuring the intensity of detected bands compared to intact tibia samples and normalized to β-actin with ImageJ software. RNA was extracted from CFU cell cultures using Trizol (Invitrogen) and cDNA was generated with Superscript II (Invitrogen). For real-time reverse transcriptase PCR (RT-PCR), mouse–specific Taq-Man fluorogenic probes for alkaline phosphatase, collagen type I, osteocalcin, and osteopontin were used. Asparagine synthetase and glyceraldehyde-3-phosphate were used as internal control genes.

### Cell culture

Unfractured tibia of 20-month old mice was isolated and contents of medullary cavity (bone marrow) were flushed using αMEM culture medium (Gibco) on POD 21 days. Cell suspensions were passed through an 18G needle to dissociate cell aggregates, and cells were plated in 24-well plates with a density of 5 × 10^5^ cells/ml in plating medium (αMEM, 10% FBS, 1% Penicillin/Streptomycin) or 1 × 10^6^ cells/ml for seven days. To determine the colony forming units-fibroblastic after14 days with plating media; wells were then washed with PBS, fixed with 10% formalin, and stained with 0.05% crystal violet. Cells were cultured in osteogenic differentiation media (αMEM, 10% FBS, 1% Penicillin/Streptomycin, 30 uM ascorbic acid, 10^–8^ M dexamethasone, 8 mM sodium phosphate) for next 14 days. Cultures were incubated for 21 days at 37 °C with 5% CO_2_ and media were replenished every 2 days. Wells were washed with PBS, fixed using 10% formalin, and stained for alkaline phosphatase (ALP) using NBT/BCIP and mineralization using 2.5% silver nitrate solution (Von Kossa) on a light box and 2% Alizarin Red S (pH 4.3). Colonies were defined by containing at least 25 cells.

### Mechanical testing to determine work to failure

Tibiae for mechanical testing were harvested four weeks following the generation of a Fracture. Intact tibiae were harvested, soft tissue removed, and immediately wrapped in PBS soaked gauze and then frozen at −20 °C. The limbs were thawed prior to mechanical testing. This process has been shown to be the best method of preservation of mechanical properties in bone. The inherent variability in mechanical testing was found to be greater than any loss in bone properties due to the processing^23–25^. Tibia were tested to failure in four-point bending on a Precision Materials testing machine (Acumen 3 A/T, MTS, Eden Prairie, MN) with a 500N force transducer (model 661.11H.02, MTS, Eden Prairie, MN). The supports of the four-point bend fixture were cylindrical and 2 mm in diameter. The distance between the midpoints of the lower load supports was set to 8.9 mm, and the distance between the midpoints of the upper load supports was set to 4 mm, spanning the fracture callus and centered over the lower supports. Tibiae were loaded by a flexion moment with the flat anteromedial surface down^26^ on the lower supports at a constant displacement rate of 0.03 mm/s^27^ to failure. The data acquisition rate for load, displacement, and time was 20 Hz. Failure loads were calculated using a peak-prominence calculation in MatLab. A prominence of 0.25 was used for intact contralateral tibia and 0.4 for fractured tibia. Failure loads were then confirmed visually as the primary departure of a linear trend of the load-displacement data. Bending stiffness was calculated as the slope of the load v. displacement data between 30–70% of the load at failure to exclude the nonlinear toe-regions. Energy to failure (mJ) was calculated using a trapezoidal algorithm of the area under the load-displacement curves.

### Statistical analysis and data sharing

A priori power analysis to obtain statistical significance (p = 0.05, power 80%) between the two groups. Sample sizes were determined using a power calculation based on variances from prior investigations: Micro-computed-tomography (μCT) imaging, n ≥ 7 per group on day 21; histology, n ≥ 7 per group on day 21; colony forming unit (CFU), n ≥ 3 per group on days 21; western blot, n ≥ 3 per group on days 3. μCT and histology data were analyzed in a blinded manner. Data are expressed as the means ± 95% confidence intervals. The Bartlett test was used to determine if the comparison groups followed a normal distribution. If the Bartlett test showed that the variances equal statistical significance was evaluated through a Student’s t-test for all comparisons in which there were two groups; or a one-way analysis of variance (ANOVA) followed by a Turkey post hoc testing for analyses in which there were three or more comparisons being made. If Bartlett test showed that the variances were not parametric, the Kruskal-Wallis test to test for differences in population means instead of ANOVA and the Steel-Dwass test was used instead of Turkey^28,29^. The data shown in Figs 1D and 4B was analyzed using a non-parametric approach. The data differences were considered significant at p < 0.05. GraphPad software was used for analysis (GraphPad Software, San Diego, CA). The datasets generated during and/or analyzed during the current study are available from the corresponding author on reasonable request.

## Supplementary information

supplementary information
