# Supplementary material for: Pharmacologic targeting of β-catenin improves fracture healing in old mice
Source: Sci Rep. 2019 Jun 21;9:9005. doi: 10.1038/s41598-019-45339-0 (PMC6588693; doi:10.1038/s41598-019-45339-0)
Supplement: Supplementary file 1 — supplementary information [file 41598_2019_45339_MOESM1_ESM.pdf]

Pharmacologic targeting of β-catenin improves fracture healing in old mice

Supplementary data

Yoon Hae Kwak, Tomasa Barrientos, Bridgette Furman, Hongyuan Zhang, Vijitha Puviindran, Hattie Cutcliffe, Jonas Herfarth, Eugene Nwankwo, and Benjamin A. Alman

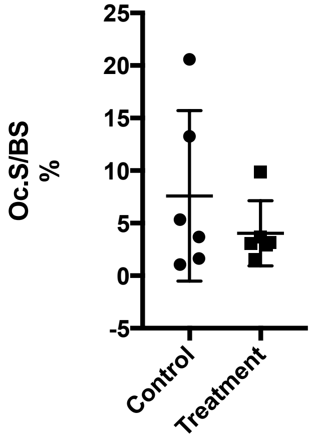

Supplementary figure one:

Osteoclast number is not affected by tankyrase inhibition.

Graphical representation of osteoclast number per bone surface. Data point from individual mice are shown as well as means and 95% confidence intervals for the animals in the treatment and control group.

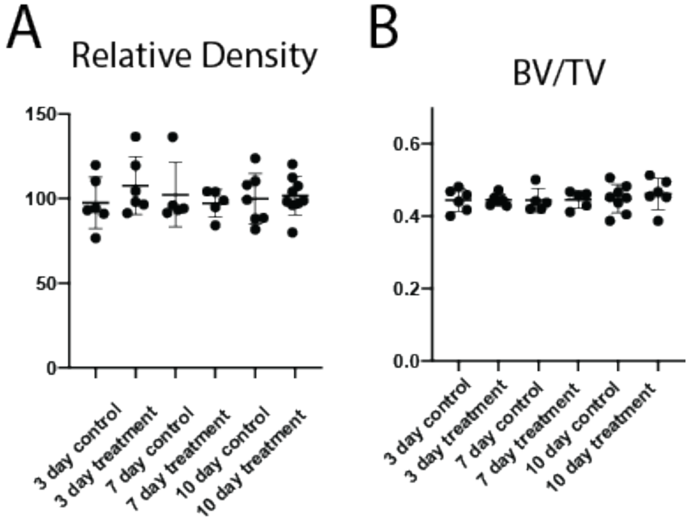

Supplementary figure two:

Nefopam treatment does not alter micro-CT characteristics of the unfractured tibia. Relative density and bone volume/total volume is shown. Each data point is shown and bars show the mean and 95% confidence intervals. There are no significant differences between any treatment or control groups.

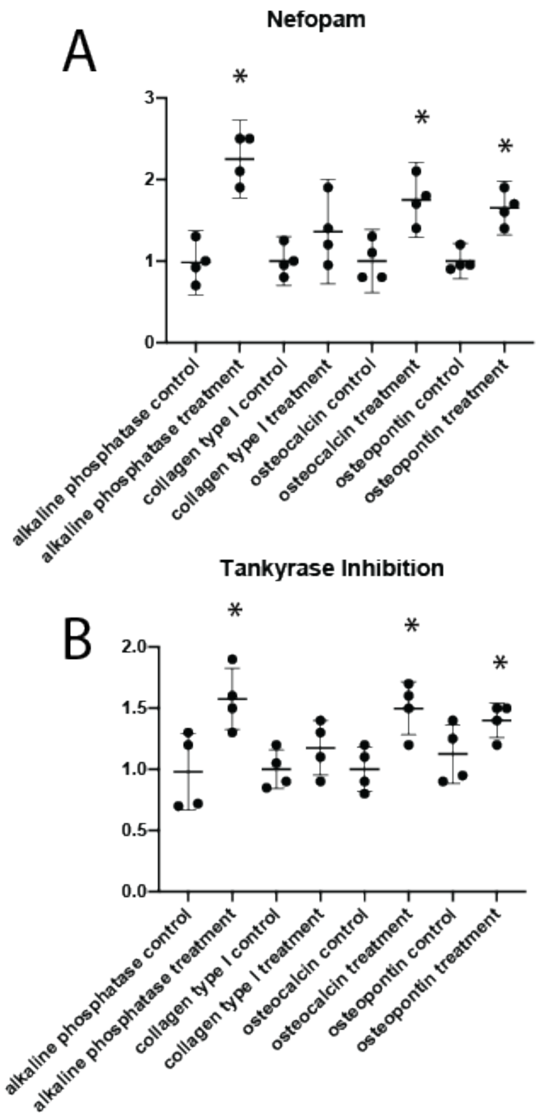

Supplementary figure three

Nefopam treatment and tankyrase inhibition increase the expression of markers of osteogenesis in CFU cultures. Each data point is shown and bars show the mean and 95% confidence intervals. Data for controls is normalized to “1”. An asterisk indicated a significant difference $p<0.05$, compared to the relevant control. Panel A is data for nefopam, panel B for tankyrase inhibition.

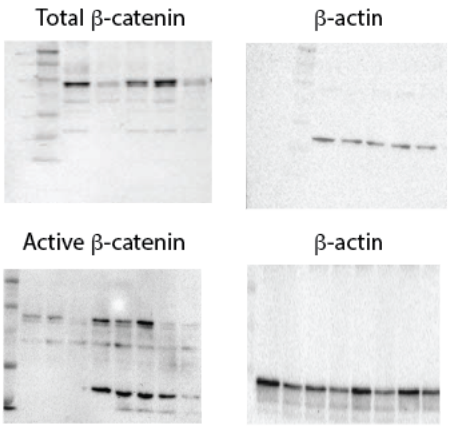

Supplementary figure four:

Full blots for the Western analysis shown in figure two.
